# The role of endoscopic ultrasound in the evaluation of rectal cancer

**DOI:** 10.1186/1477-7800-3-36

**Published:** 2006-10-18

**Authors:** Ali A Siddiqui, Yomi Fayiga, Sergio Huerta

**Affiliations:** 1 Division of Gastroenterology, VA North Texas Health Care System and University of Texas Southwestern Medical School, Dallas, TX75216, USA; 2 Division of GI/Endocrine Surgery^2^, VA North Texas Health Care System and University of Texas Southwestern Medical School, Dallas, TX75216, USA

## Abstract

Accurate staging of rectal cancer is essential for selecting patients who can undergo sphincter-preserving surgery.  It may also identify patients who could benefit from neoadjuvant therapy. Clinical staging is usually accomplished using a combination of physical examination, CT scanning, MRI and endoscopic ultrasound (EUS). Transrectal EUS is increasingly being used for locoregional staging of rectal cancer. The accuracy of EUS for the T staging of rectal carcinoma ranges from 80-95% compared with CT (65-75%) and MR imaging (75-85%). In comparison to CT, EUS can potentially upstage patients, making them eligible for neoadjuvant treatment. The accuracy to determine metastatic nodal involvement by EUS is approximately 70-75% compared with CT (55-65%) and MR imaging (60-70%). EUS guided FNA may be beneficial in patients who appear to have early T stage disease and suspicious peri-iliac lymphadenopathy to exclude metastatic disease.

## Background

Approximately 41,000 new cases of rectal cancer will be diagnosed in the year 2006 with an estimated 8,500 deaths [1]. The prognosis and management of this malignancy is dependent upon its stage at the time of initial presentation. Previously unrecognized lymph node metastasis may present in up to 10% of T1 lesions and 17% of T2 lesions [2]. In contrast to colon cancer, clinical preoperative tumor staging is essential since it allows selection of patients in need of neoadjuvant chemoradiation and those who may benefit from tumor load reduction to facilitate resection and potentially result in sphincter-preserving resections. Neoadjuvant chemoradiation is currently recommended for patients with advanced locoregional rectal cancer, i.e. those with tumor extension into the perirectal fat and/or involvement of the mesorectal or pelvic lymph nodes (T3, T4 N0, or Tx N1, N2) [3]. In these patients, neoadjuvant therapy followed by surgery results in better local control [4], and similar or reduced toxicity when compared with standard postoperative adjuvant regimens [5]. The Swedish Rectal Cancer Trials showed that a short-term regimen of high-dose preoperative radiotherapy reduced rates of local recurrence and improved survival among patients with resectable rectal cancer [6].

Currently available methods for assessment of rectal tumors include digital rectal examination, rigid proctoscopy, computer tomography (CT) scan, magnetic resonance imaging (MRI), and endorectal ultrasound (EUS). Digital examination allows for assessment of size and degree of fixation of rectal tumors. It has limited value because of its subjective nature and dependence on the examiner's experience [7]. Rigid proctoscopy (which is usually performed in conjunction with rectal digital examination) provides the most precise assessment of tumor location and distance from the anal verge. CT scan is an excellent modality to evaluate for distant metastasis and adjacent organ involvement, but lacks specificity for loco-regional staging due to its inability to distinguish between mural layers. The staging accuracy for CT scan for rectal cancer is approximately 75% [8]. The rigid transrectal ultrasound (TRUS) is a diagnostic modality for pre-operative staging of mid and distal rectal cancers (i.e. tumors within 10 cm from the anal verge). TRUS makes it possible for assessment of bowel thickness involvement and lymph node status. MRI has a similar accuracy to TRUS and appears to be superior in more advanced lesions [9-11]. For technical reasons a rigid transrectal US (TRUS) is less feasible for evaluation for more proximal rectal cancers.

Transrectal endoscopic ultrasound has emerged as the diagnostic modality of choice for clinical staging of rectal tumors. Because EUS can delineate the layers of the rectal wall [12-14], it is superior to CT in staging accuracy. EUS and MRI can be used as complementary methods in the preoperative staging of rectal cancer. EUS is more accurate in determining bowel wall penetration of the tumor, while MRI is comparable to EUS in the evaluation of lymph node involvement [15,16]. In the present report, we review the current status of EUS in the diagnosis and staging for rectal cancer.

### Technique of rectal EUS

**Figure 1:**
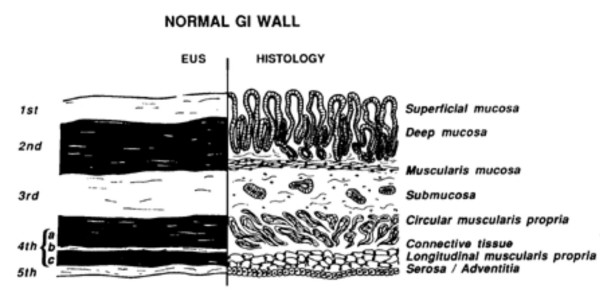
Correlation between the standard five EUS layers and histological layers of the normal intestinal wall. 1st = interface between fluid in the lumen and the superficial mucosa; 2nd = lamina propria and muscularis mucosa, or deep mucosa; 3rd = submucosa and interface between submucosa and muscularis propria; 4th = muscularis propria; circular (4a) and longitudinal (4c) are not usually seen as separate layers; 5th = interface between serosa and surrounding adventitial tissue.

The most widely used EUS endoscopes are available in two different designs: radial and curved linear array. Radial echoendoscopes produce a 360° image in a plane perpendicular to the long axis of the endoscope's insertion tube. Linear devices, on the other hand, produce sector-shaped images in a plane parallel to the long axis of the insertion tube. Linear imaging is used for interventional EUS-fine needle aspiration (FNA). EUS sonographic layers of the wall of the GI tract have been correlated to histopathological layers in several studies [17]. The standard five layer EUS image of the GI tract wall and its correlation to histological layers is shown in Figure 1.

**Figure 2:**
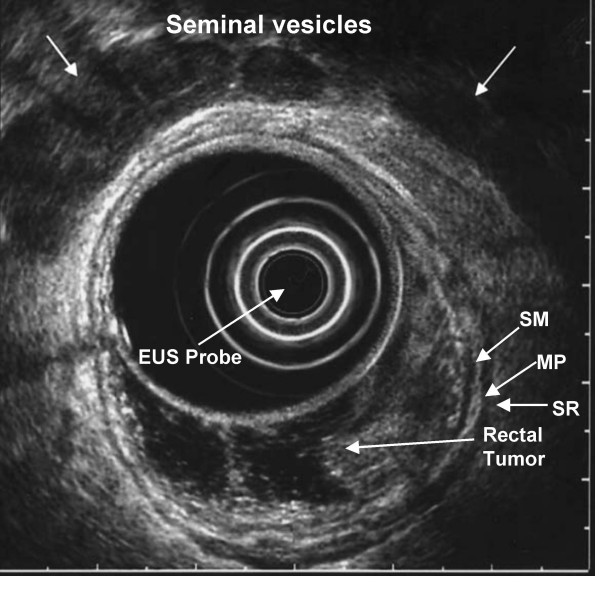
EUS image of T1 rectal cancer confined to mucosa and superficial submucosa. SM=submucosa, MP=muscularis propria,  SR=serosa.

Patients who undergo rectal EUS should receive an oral lavage preparation used typically consisting of polyethylene glycol electrolyte solution. Tumor staging and lymph node detection is performed by filling the echoendoscope ultrasound balloon with water, advancing the scope above the tumor and slowly withdrawing to the anal sphincters. The depth of wall invasion, invasion into the perirectal fat or adjacent organs, and presence of perirectal lymph nodes are carefully assessed (Figure 2). If a node is enlarged, EUS may be used to proceed with fine needle aspiration (FNA) sampling of suspicious lesions (perirectal or iliac lymphadenopathy).

### Staging of rectal cancer

Prognosis of rectal cancer depends on its local, nodal, and distant tumor status. Rectal cancer is staged using the Tumor-Node-Metastasis (TNM) staging system, which is similar to colon cancer (Table 1). Rectal cancer has the appearance of a hypoechoic lesion which disrupts the normal five-layer structure of the rectal wall. The accuracy of EUS for the T staging of rectal carcinoma ranges from 80–95% [13,18] compared with CT (65–75%) and MR imaging (75–85%) [13,18-20].

**Table 1:** AJCC TNM Rectal Cancer Staging

TX	Primary tumor can not be assessed.
T0	No primary tumor identified.
Tis	Carcinoma in situ (tumor limited to mucosa).
T1	Involvement of submucosa, but no penetration through muscularis propria.
T2	Invasion into, but not penetration through, muscularis propria.
T3	Penetration through muscularis propria into subserosa (if present), or pericolic fat, but not into peritoneal cavity or other organs.
T4	Invasion of other organs or involvement of free peritoneal cavity.
NX	Nodal metastasis can not be assessed.
N0	No nodal metastasis.
N1	1–3 pericolic/perirectal nodes involved.
N2	4 or more pericolic/perirectal nodes involved.
MX	Distant metastasis can not be assessed.
M0	No distant metastases.
M1	Distant metastases

**Figure 3:**
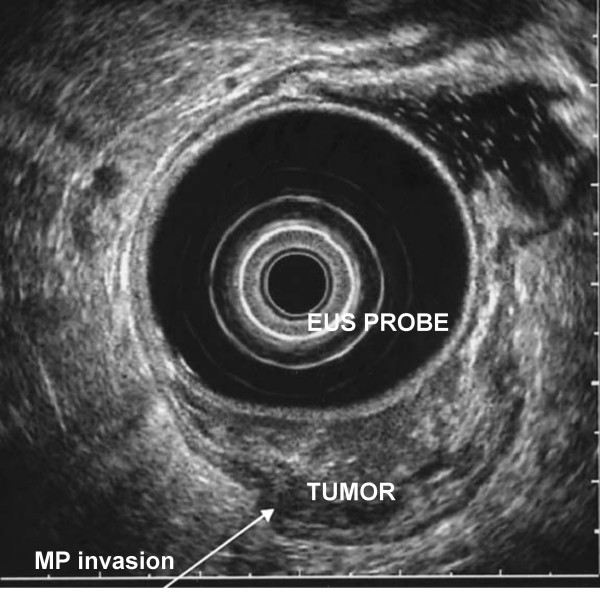
EUS image of T2 rectal cancer invading the muscularis propria.

One of the limitations of EUS is the under-staging of T3 tumors, which is caused by the inability to detect microscopic cancer infiltration owing to the limits its resolution. Other factors influencing the accuracy of tumor staging are operator experience [8,21,22] and the level of the tumor, with reduced accuracy for tumors lower in the rectum [8,23]. Staging accuracy is best for T2 tumors (Figure 3) [24]. However, over-staging of T2 tumors may occur as a result of inflammation around the tumor which is sonographically indistinguishable from malignant tissue [25]. Circumferential rectal tumors causing stenosis may not be able to be staged accurately because of inability to traverse the stenosis by the endoscope [26]. Additionally, preoperative radiotherapy decreases the EUS accuracy for T stage interpretation because of the increased echogenicity of the rectal wall resulting from radiation therapy [27].

**Figure 4:**
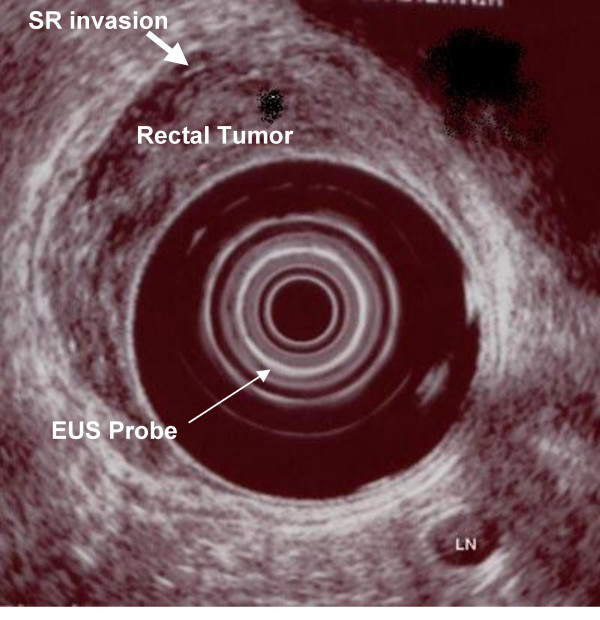
EUS image of T4 rectal cancer with a metastasis to a perirectal lymph node (L).

The accuracy to determine metastatic nodal involvement by EUS is approximately 70–75% [18,19,28] compared with CT (55–65%) [20,29] and MR imaging (60–70%) [19,30]. Nodal staging by EUS is less accurate than tumor staging due to difficulty of detecting tumor within a lymph node. Lymph node changes associated with malignant involvement by EUS include a hypoechoic appearance, round shape of the node, and nodal diameter of 1 cm or greater (Figure 4) [25,27,31]. A rectal cancer with a malignant perirectal lymph node (L) is demonstrated in Figure 4. Lymph nodes greater than 0.5 cm in diameter have a 50–70% possibility of being metastatic, whereas those smaller than 4 mm have a less than 20% likelihood [7].

EUS-guided FNA does not improve the preoperative staging of rectal cancer in most patients. Harewood *et al *evaluated the role of EUS-guided FNA of perirectal lymph nodes in the preoperative assessment of a cohort of 80 patients with newly diagnosed, nonmetastatic rectal cancer [32]. The overall accuracy for N staging was similar (80%) for CT, EUS, and EUS FNA with no benefit in accuracy from performing FNA. FNA may not significantly improve nodal involvement accuracy compared to EUS alone because perirectal lymph nodes are usually too small to be visualized by EUS unless they contain metastatic disease.

The overall accuracy of EUS, CT scan and MRI in the staging rectal cancer compared with surgical pathology is summarized in table 2.

**Table 2:** Accuracy of rectal US in staging rectal cancer compared with surgical pathology

	**T-Staging**	**N-Staging**
**EUS**	80–95% [13;18]	70–75% [18;19;28]
**Helical CT**	65–75% [13;18-20]	55–65% [20;29]
**MRI**	75–85% [13;18-20]	60–70% [19;30]

### The impact of rectal EUS on management

The routine utilization of EUS for assessment and determination of tumor penetration into the bowel wall is essential for rectal cancer. This has become the standard of care as it allows for identification of patients in whom to administer preoperative neoadjuvant therapy [32]. Analysis of a cohort of 80 patients with diagnosed rectal cancer showed that EUS was more accurate than CT for determining T stage of rectal carcinoma. The addition of rectal EUS to the diagnostic work-up resulted in a change in the number of patients receiving preoperative adjuvant therapy in nearly one third of these patient, which reflects the accuracy of EUS compared to CT scan or MRI. On the other hand, it is possible that the aggressive use of EUS may lead to over staging and over treating of rectal tumors which would typically only need resection. However, the positive predictive value (PPV) for identifying T3/T4 disease by EUS was 100%. Additionally, rectal EUS can detect patients with advanced T stage disease missed by CT who will benefit from preoperative treatment. Thus, the risk benefit ratio of over staging is justified. Even with aggressive EUS practices, EUS understaged 15% of those patients in whom neoadjuvant therapy would typically be considered. Yet, this was lower than CT scan, which understage tumors in 39% of patients [32]. Additionally, cost-effectiveness analysis of 3 different staging strategies (abdominal and pelvic CT versus abdominal CT plus EUS versus abdominal CT plus pelvic MRI) found that abdominal CT plus EUS is the most cost-effective approach for nonmetastatic proximal rectal cancer [33]. The correlation of staging by EUS with the treatment protocol for rectal cancer is summarized in Table 3.

**Table 3:** Correlation of staging with recommended treatment for rectal cancer

**EUS STAGE**	**HISTOPATHOLOGICAL STAGING**	**THERAPY**
**UT1**	Invasion of mucosa and submucosa	Excision
**UT2**	Invasion of tumor into the muscularis propia	Excision
**UT3**	Invasion of tumor through the serosa	Pre-op chemoradiotherapy → resection of tumor
**UT4**	Invasion of tumor into adjacent organs	Pre-op chemoradiotherapy → resection of tumor → post-op chemotherapy

### Rectal EUS staging after radiation therapy

Accuracy of EUS for staging rectal cancer after radiation therapy is decreased markedly due to post-radiation edema, inflammation, necrosis, and fibrosis. Studies suggest that the T-stage accuracy after radiation is 50%, with a 40% overstaging rate [27,34]. Lymph node staging accuracy is also decreased. Thus, at this juncture, restaging tumors after neoadjuvant therapy is limited and clinical correlation is most important to dictate operative and postoperative management modalities.

### Recurrent rectal cancer

Local recurrence rate after surgery alone for advanced rectal cancer is approximately 25% and decreases to 10% after radiation [33]. The risk of recurrence is greatest in the first 2 years after surgery. Early detection of recurrent local tumor might result in earlier treatment and improved survival. The ability of CT scan is limited because if may not be able to distinguishing recurrence from postoperative change due to fibrosis or inflammation [35]. CT images can also be obscured by artifacts from surgically placed metal clips. Two prospective studies demonstrated that EUS was superior to CT scan for local recurrence detection in rectal cancer [35,36]. The sensitivity of EUS for detecting recurrence was higher (100 percent) in both studies compared to CT (82 to 85 percent). EUS was also more sensitive than digital rectal examination, CT and CEA levels to detect local recurrence of rectal cancer in patient who were asymptomatic from their disease [37]. EUS specificity is limited by its inability to distinguish between mucosal inflammation and recurrence. Although the optimal interval for repeating EUS after surgical treatment of rectal cancer is unknown, performing rectal EUS every 6 months for the first 2 years after low anterior resection or transanal excision may be a reasonable screening for recurrent rectal cancer [38, 39].

## Conclusion

The use of preoperative EUS is an accurate modality for clinical staging of rectal cancer to guide neoadjuvant treatment. EUS guided-FNA may be beneficial in patients who appear to have early T stage disease and suspicious peri-iliac lymphadenopathy. Whether the accurate staging ability of EUS and EUS guided-FNA translates into improved outcomes in terms of reduced recurrence rates and ultimately prolonged survival remains uncertain. At this juncture, the utility of FNA-guided needle aspiration for evaluation of metastatic disease remains unclear.

## Competing interests

The author(s) declare that they have no competing interests.

## Authors' contributions

AAS conceived of the idea of the review article, and helped to draft the manuscript. YF helped to draft the manuscript. SH coordinated and helped to draft the manuscript. All authors read and approved the final manuscript.

## References

[B1] Jemal A, Siegel R, Ward E, Murray T, Xu J, Smigal C, Thun MJ (2006). Cancer statistics, 2006. CA Cancer J Clin.

[B2] Blumberg D, Paty PB, Guillem JG, Picon AI, Minsky BD, Wong WD, Cohen AM (1999). All patients with small intramural rectal cancers are at risk for lymph node metastasis. Dis Colon Rectum.

[B3] Crane CH, Skibber J (2003). Preoperative chemoradiation for locally advanced rectal cancer: rationale, technique, and results of treatment. Semin Surg Oncol.

[B4] Minsky BD (1997). Adjuvant therapy for rectal cancer--a good first step. N Engl J Med.

[B5] Grann A, Feng C, Wong D, Saltz L, Paty PP, Guillem JG, Cohen AM, Minsky BD (2001). Preoperative combined modality therapy for clinically resectable uT3 rectal adenocarcinoma. Int J Radiat Oncol Biol Phys.

[B6] Pahlman L, Glimelius B (1990). Pre- or postoperative radiotherapy in rectal and rectosigmoid carcinoma. Report from a randomized multicenter trial. Ann Surg.

[B7] Beynon J, Mortensen NJ, Foy DM, Channer JL, Virjee J, Goddard P (1986). Pre-operative assessment of local invasion in rectal cancer: digital examination, endoluminal sonography or computed tomography?. Br J Surg.

[B8] Herzog U, von FM, Tondelli P, Schuppisser JP (1993). How accurate is endorectal ultrasound in the preoperative staging of rectal cancer?. Dis Colon Rectum.

[B9] Fleshman JW, Myerson RJ, Fry RD, Kodner IJ (1992). Accuracy of transrectal ultrasound in predicting pathologic stage of rectal cancer before and after preoperative radiation therapy. Dis Colon Rectum.

[B10] Mathur P, Smith JJ, Ramsey C, Owen M, Thorpe A, Karim S, Burke C, Ramesh S, Dawson PM (2003). Comparison of CT and MRI in the pre-operative staging of rectal adenocarcinoma and prediction of circumferential resection margin involvement by MRI. Colorectal Dis.

[B11] Beets-Tan RG (2003). MRI in rectal cancer: the T stage and circumferential resection margin. Colorectal Dis.

[B12] Kwok H, Bissett IP, Hill GL (2000). Preoperative staging of rectal cancer. Int J Colorectal Dis.

[B13] Waizer A, Powsner E, Russo I, Hadar S, Cytron S, Lombrozo R, Wolloch Y, Antebi E (1991). Prospective comparative study of magnetic resonance imaging versus transrectal ultrasound for preoperative staging and follow-up of rectal cancer. Preliminary report. Dis Colon Rectum.

[B14] Meyenberger C, Huch Boni RA, Bertschinger P, Zala GF, Klotz HP, Krestin GP (1995). Endoscopic ultrasound and endorectal magnetic resonance imaging: a prospective, comparative study for preoperative staging and follow-up of rectal cancer. Endoscopy.

[B15] Bianchi PP, Ceriani C, Rottoli M, Torzilli G, Pompili G, Malesci A, Ferraroni M, Montorsi M (2005). Endoscopic ultrasonography and magnetic resonance in preoperative staging of rectal cancer: comparison with histologic findings. J Gastrointest Surg.

[B16] Bianchi P, Ceriani C, Palmisano A, Pompili G, Passoni GR, Rottoli M, Cappellani A, Montorsi M (2006). A prospective comparison of endorectal ultrasound and pelvic magnetic resonance in the preoperative staging of rectal cancer. Ann Ital Chir.

[B17] Snady H (1994). Role of endoscopic ultrasonography in diagnosis, staging, and outcome of gastrointestinal diseases. Gastroenterologist.

[B18] Tio TL, Coene PP, van Delden OM, Tytgat GN (1991). Colorectal carcinoma: preoperative TNM classification with endosonography. Radiology.

[B19] Thaler W, Watzka S, Martin F, La GG, Psenner K, Bonatti G, Fichtel G, Egarter-Vigl E, Marzoli GP (1994). Preoperative staging of rectal cancer by endoluminal ultrasound vs. magnetic resonance imaging. Preliminary results of a prospective, comparative study. Dis Colon Rectum.

[B20] Guinet C, Buy JN, Ghossain MA, Sezeur A, Mallet A, Bigot JM, Vadrot D, Ecoiffier J (1990). Comparison of magnetic resonance imaging and computed tomography in the preoperative staging of rectal cancer. Arch Surg.

[B21] Orrom WJ, Wong WD, Rothenberger DA, Jensen LL, Goldberg SM (1990). Endorectal ultrasound in the preoperative staging of rectal tumors. A learning experience. Dis Colon Rectum.

[B22] Glaser F, Schlag P, Herfarth C (1990). Endorectal ultrasonography for the assessment of invasion of rectal tumours and lymph node involvement. Br J Surg.

[B23] Sailer M, Leppert R, Bussen D, Fuchs KH, Thiede A (1997). Influence of tumor position on accuracy of endorectal ultrasound staging. Dis Colon Rectum.

[B24] Starck M, Bohe M, Simanaitis M, Valentin L (2003). Rectal endosonography can distinguish benign rectal lesions from invasive early rectal cancers. Colorectal Dis.

[B25] Hulsmans FH, Bosma A, Mulder PJ, Reeders JW, Tytgat GN (1992). Perirectal lymph nodes in rectal cancer: in vitro correlation of sonographic parameters and histopathologic findings. Radiology.

[B26] Hawes RH (1993). New staging techniques. Endoscopic ultrasound. Cancer.

[B27] Napoleon B, Pujol B, Berger F, Valette PJ, Gerard JP, Souquet JC (1991). Accuracy of endosonography in the staging of rectal cancer treated by radiotherapy. Br J Surg.

[B28] Hildebrandt U, Klein T, Feifel G, Schwarz HP, Koch B, Schmitt RM (1990). Endosonography of pararectal lymph nodes. In vitro and in vivo evaluation. Dis Colon Rectum.

[B29] Rifkin MD, Ehrlich SM, Marks G (1989). Staging of rectal carcinoma: prospective comparison of endorectal US and CT. Radiology.

[B30] Saitoh N, Okui K, Sarashina H, Suzuki M, Arai T, Nunomura M (1986). Evaluation of echographic diagnosis of rectal cancer using intrarectal ultrasonic examination. Dis Colon Rectum.

[B31] Harewood GC, Wiersema MJ, Nelson H, Maccarty RL, Olson JE, Clain JE, Ahlquist DA, Jondal ML (2002). A prospective, blinded assessment of the impact of preoperative staging on the management of rectal cancer. Gastroenterology.

[B32] Harewood GC, Wiersema MJ (2002). Cost-effectiveness of endoscopic ultrasonography in the evaluation of proximal rectal cancer. Am J Gastroenterol.

[B33] Rau B, Hunerbein M, Barth C, Wust P, Haensch W, Riess H, Felix R, Schlag PM (1999). Accuracy of endorectal ultrasound after preoperative radiochemotherapy in locally advanced rectal cancer. Surg Endosc.

[B34] Ramirez JM, Mortensen NJ, Takeuchi N, Humphreys MM (1994). Endoluminal ultrasonography in the follow-up of patients with rectal cancer. Br J Surg.

[B35] Novell F, Pascual S, Viella P, Trias M (1997). Endorectal ultrasonography in the follow-up of rectal cancer. Is it a better way to detect early local recurrence?. Int J Colorectal Dis.

[B36] Lohnert M, Dohrmann P, Stoffregen C, Hamelmann H (1991). [Value of endorectal sonography in the follow-up of patients treated surgically for rectum carcinoma]. Zentralbl Chir.

[B37] Mellgren A, Sirivongs P, Rothenberger DA, Madoff RD, Garcia-Aguilar J (2000). Is local excision adequate therapy for early rectal cancer?. Dis Colon Rectum.

